# Supplementary material for: Regulation of telomere silencing by the core histones–autophagy–Sir2 axis
Source: Life Sci Alliance. 2022 Dec 30;6(3):e202201614. doi: 10.26508/lsa.202201614 (PMC9806677; doi:10.26508/lsa.202201614)
Supplement: Supplementary file 9 [file LSA-2022-01614_TableS1.docx]

**Supplemental Table 1 List of strains used in this study**

| **Name Parental** | **Strain** | **Genotype** | **Source** |
| --- | --- | --- | --- |
| BY4741 | BY4741 | *MATa his3∆1 leu2∆0 met15∆0 ura3∆0* | This study |
| H3/H4 KD | BY4741 | *MATa his3∆1 leu2∆0 met15∆0 ura3∆0 hhf2-hht2::MET15 hhf1-hht1::LEU2, pDM9-HHT1-HHF1-URA3* | This study |
| TEL WTH3 | UCC1369 | *MATa ade2::hisG his3Δ200 leu2Δ0 lys2Δ0 met15Δ0 trp1Δ63 ura3Δ0 adh4::URA3-TEL-VIIL AdE2-TEL-VR, hhf2-hht2::MET15 hhf1-hht1::LEU2, pDM18-HHT2-HHF2-TRP1* | This study |
| *Atg2∆* | BY4741 | *MATa his3∆1 leu2∆0 met15∆0 ura3∆0 atg2∆::KAN* | This study |
| *atg12∆* | BY4741 | *MATa his3∆1 leu2∆0 met15∆0 ura3∆0 atg12∆::KAN* | This study |
| *hir1∆* | BY4741 | *MATa his3∆1 leu2∆0 met15∆0 ura3∆0 hir1∆::KAN* | This study |
| *hir2∆* | BY4741 | *MATa his3∆1 leu2∆0 met15∆0 ura3∆0 hir2∆::KAN* | This study |
| *hir3∆* | BY4741 | *MATa his3∆1 leu2∆0 met15∆0 ura3∆0 hir3∆::KAN* | This study |
| *hpc2∆* | BY4741 | *MATa his3∆1 leu2∆0 met15∆0 ura3∆0 hpc2∆::KAN* | This study |
| *set1∆* | BY4741 | *MATa his3∆1 leu2∆0 met15∆0 ura3∆0 set1∆::KAN* | This study |
| *spp1∆* | BY4741 | *MATa his3∆1 leu2∆0 met15∆0 ura3∆0 spp1∆::KAN* | This study |
| *spt21∆* | BY4741 | *MATa his3∆1 leu2∆0 met15∆0 ura3∆0 spt21∆::KAN* | This study |
| *sir2∆* | BY4741 | *MATa his3∆1 leu2∆0 met15∆0 ura3∆0 sir2∆::KAN* | This study |
| *set1∆ atg12∆* | BY4741 | *MATa his3∆1 leu2∆0 met15∆0 ura3∆0 set1∆::KAN atg12∆::HIS3* | This study |
| H3/H4 KD *atg2∆* | BY4741 | *MATa his3∆1 leu2∆0 met15∆0 ura3∆0 hhf2-hht2::MET15 hhf1-hht1::LEU2, pDM9-HHT1-HHF1-URA3 atg2∆::HIS3* | This study |
| H3/H4 KD *atg12∆* | BY4741 | *MATa his3∆1 leu2∆0 met15∆0 ura3∆0 hhf2-hht2::MET15 hhf1-hht1::LEU2, pDM9-HHT1-HHF1-URA3 atg12∆::HIS3* | This study |
| *spt21∆* | UCC1369 | *MATa ade2::hisG his3Δ200 leu2Δ0 lys2Δ0 met15Δ0 trp1Δ63 ura3Δ0 adh4::URA3-TEL-VIIL AdE2-TEL-VR, hhf2-hht2::MET15 hhf1-hht1::LEU2, pDM18-HHT2-HHF2-TRP1 spt21∆::HIS3* | This study |
| H3R2A | UCC1369 | *MATa ade2::hisG his3Δ200 leu2Δ0 lys2Δ0 met15Δ0 trp1Δ63 ura3Δ0 adh4::URA3-TEL-VIIL AdE2-TEL-VR, hhf2-hht2::MET15 hhf1-hht1::LEU2, pDM18-HHT2(R2A)-HHF2-TRP1* | This study |
| H3K4A | UCC1369 | *MATa ade2::hisG his3Δ200 leu2Δ0 lys2Δ0 met15Δ0 trp1Δ63 ura3Δ0 adh4::URA3-TEL-VIIL AdE2-TEL-VR, hhf2-hht2::MET15 hhf1-hht1::LEU2, pDM18-HHT2(K4A)-HHF2-TRP1* | This study |
| H3K4M | UCC1369 | *MATa ade2::hisG his3Δ200 leu2Δ0 lys2Δ0 met15Δ0 trp1Δ63 ura3Δ0 adh4::URA3-TEL-VIIL AdE2-TEL-VR, hhf2-hht2::MET15 hhf1-hht1::LEU2, pDM18-HHT2(K4M)-HHF2-TRP1* | This study |
| H3K4R | UCC1369 | *MATa ade2::hisG his3Δ200 leu2Δ0 lys2Δ0 met15Δ0 trp1Δ63 ura3Δ0 adh4::URA3-TEL-VIIL AdE2-TEL-VR, hhf2-hht2::MET15 hhf1-hht1::LEU2, pDM18-HHT2(K4R)-HHF2-TRP1* | This study |
| H3T6A | UCC1369 | *MATa ade2::hisG his3Δ200 leu2Δ0 lys2Δ0 met15Δ0 trp1Δ63 ura3Δ0 adh4::URA3-TEL-VIIL AdE2-TEL-VR, hhf2-hht2::MET15 hhf1-hht1::LEU2, pDM18-HHT2(T6A)-HHF2-TRP1* | This study |
| H3K14A | UCC1369 | *MATa ade2::hisG his3Δ200 leu2Δ0 lys2Δ0 met15Δ0 trp1Δ63 ura3Δ0 adh4::URA3-TEL-VIIL AdE2-TEL-VR, hhf2-hht2::MET15 hhf1-hht1::LEU2, pDM18-HHT2(K14A)-HHF2-TRP1* | This study |
| H3R17A | UCC1369 | *MATa ade2::hisG his3Δ200 leu2Δ0 lys2Δ0 met15Δ0 trp1Δ63 ura3Δ0 adh4::URA3-TEL-VIIL AdE2-TEL-VR, hhf2-hht2::MET15 hhf1-hht1::LEU2, pDM18-HHT2(R17A)-HHF2-TRP1* | This study |
| H3R40A | UCC1369 | *MATa ade2::hisG his3Δ200 leu2Δ0 lys2Δ0 met15Δ0 trp1Δ63 ura3Δ0 adh4::URA3-TEL-VIIL AdE2-TEL-VR, hhf2-hht2::MET15 hhf1-hht1::LEU2, pDM18-HHT2(R40A)-HHF2-TRP1* | This study |
| H3R49A | UCC1369 | *MATa ade2::hisG his3Δ200 leu2Δ0 lys2Δ0 met15Δ0 trp1Δ63 ura3Δ0 adh4::URA3-TEL-VIIL AdE2-TEL-VR, hhf2-hht2::MET15 hhf1-hht1::LEU2, pDM18-HHT2(R49A)-HHF2-TRP1* | This study |
| H3R53A | UCC1369 | *MATa ade2::hisG his3Δ200 leu2Δ0 lys2Δ0 met15Δ0 trp1Δ63 ura3Δ0 adh4::URA3-TEL-VIIL AdE2-TEL-VR, hhf2-hht2::MET15 hhf1-hht1::LEU2, pDM18-HHT2(R53A)-HHF2-TRP1* | This study |
| H3K56A | UCC1369 | *MATa ade2::hisG his3Δ200 leu2Δ0 lys2Δ0 met15Δ0 trp1Δ63 ura3Δ0 adh4::URA3-TEL-VIIL AdE2-TEL-VR, hhf2-hht2::MET15 hhf1-hht1::LEU2, pDM18-HHT2(K56A)-HHF2-TRP1* | This study |
| H3R69A | UCC1369 | *MATa ade2::hisG his3Δ200 leu2Δ0 lys2Δ0 met15Δ0 trp1Δ63 ura3Δ0 adh4::URA3-TEL-VIIL AdE2-TEL-VR, hhf2-hht2::MET15 hhf1-hht1::LEU2, pDM18-HHT2(R69A)-HHF2-TRP1* | This study |
| H3R72A | UCC1369 | *MATa ade2::hisG his3Δ200 leu2Δ0 lys2Δ0 met15Δ0 trp1Δ63 ura3Δ0 adh4::URA3-TEL-VIIL AdE2-TEL-VR, hhf2-hht2::MET15 hhf1-hht1::LEU2, pDM18-HHT2(R72A)-HHF2-TRP1* | This study |
| H3D77A | UCC1369 | *MATa ade2::hisG his3Δ200 leu2Δ0 lys2Δ0 met15Δ0 trp1Δ63 ura3Δ0 adh4::URA3-TEL-VIIL AdE2-TEL-VR, hhf2-hht2::MET15 hhf1-hht1::LEU2, pDM18-HHT2(D77A)-HHF2-TRP1* | This study |
| H3D81A | UCC1369 | *MATa ade2::hisG his3Δ200 leu2Δ0 lys2Δ0 met15Δ0 trp1Δ63 ura3Δ0 adh4::URA3-TEL-VIIL AdE2-TEL-VR, hhf2-hht2::MET15 hhf1-hht1::LEU2, pDM18-HHT2(D81A)-HHF2-TRP1* | This study |
| H3Q85A | UCC1369 | *MATa ade2::hisG his3Δ200 leu2Δ0 lys2Δ0 met15Δ0 trp1Δ63 ura3Δ0 adh4::URA3-TEL-VIIL AdE2-TEL-VR, hhf2-hht2::MET15 hhf1-hht1::LEU2, pDM18-HHT2(Q85A)-HHF2-TRP1* | This study |
| H3S87A | UCC1369 | *MATa ade2::hisG his3Δ200 leu2Δ0 lys2Δ0 met15Δ0 trp1Δ63 ura3Δ0 adh4::URA3-TEL-VIIL AdE2-TEL-VR, hhf2-hht2::MET15 hhf1-hht1::LEU2, pDM18-HHT2(S87A)-HHF2-TRP1* | This study |
| H3F104A | UCC1369 | *MATa ade2::hisG his3Δ200 leu2Δ0 lys2Δ0 met15Δ0 trp1Δ63 ura3Δ0 adh4::URA3-TEL-VIIL AdE2-TEL-VR, hhf2-hht2::MET15 hhf1-hht1::LEU2, pDM18-HHT2(F104A)-HHF2-TRP1* | This study |
| H4L37A | UCC1369 | *MATa ade2::hisG his3Δ200 leu2Δ0 lys2Δ0 met15Δ0 trp1Δ63 ura3Δ0 adh4::URA3-TEL-VIIL AdE2-TEL-VR, hhf2-hht2::MET15 hhf1-hht1::LEU2, pDM18-HHT2-HHF2(L37A)-TRP1* | This study |
| H4K44A | UCC1369 | *MATa ade2::hisG his3Δ200 leu2Δ0 lys2Δ0 met15Δ0 trp1Δ63 ura3Δ0 adh4::URA3-TEL-VIIL AdE2-TEL-VR, hhf2-hht2::MET15 hhf1-hht1::LEU2, pDM18-HHT2-HHF2(K44A)-TRP1* | This study |
| H4R55A | UCC1369 | *MATa ade2::hisG his3Δ200 leu2Δ0 lys2Δ0 met15Δ0 trp1Δ63 ura3Δ0 adh4::URA3-TEL-VIIL AdE2-TEL-VR, hhf2-hht2::MET15 hhf1-hht1::LEU2, pDM18-HHT2-HHF2(R55A)-TRP1* | This study |
| H4T96A | UCC1369 | *MATa ade2::hisG his3Δ200 leu2Δ0 lys2Δ0 met15Δ0 trp1Δ63 ura3Δ0 adh4::URA3-TEL-VIIL AdE2-TEL-VR, hhf2-hht2::MET15 hhf1-hht1::LEU2, pDM18-HHT2-HHF2(T96A)-TRP1* | This study |
| BY4741  empty vector | BY4741 | *MATa his3Δ1 leu2Δ0 met15Δ0 ura3Δ0 pESC-LEU2* | This study |
| *set1∆*  empty vector | BY4741 | *MATa his3Δ1 leu2Δ0 met15Δ0 ura3Δ0 set1∆::KAN pESC-LEU2* | This study |
| *spp1∆*  empty vector | BY4741 | *MATa his3Δ1 leu2Δ0 met15Δ0 ura3Δ0 spp1∆::KAN pESC-LEU2* | This study |
| BY4741  Gal H3/H4  (H3/H4 OE) | BY4741 | *MATa his3Δ1 leu2Δ0 met15Δ0 ura3Δ0 pESC-LEU2 -pGAL-HHT2-HHF2* | This study |
| *set1∆*  Gal H3/H4 | BY4741 | *MATa his3Δ1 leu2Δ0 met15Δ0 ura3Δ0 set1∆::KAN pESC-LEU2-pGAL-HHT2-HHF2* | This study |
| *spp1∆*  Gal H3/H4 | BY4741 | *MATa his3Δ1 leu2Δ0 met15Δ0 ura3Δ0 spp1∆::KAN pESC-LEU2-pGAL-HHT2-HHF2* | This study |
| *Spt21∆*  Gal H3/H4 | BY4741 | *MATa his3Δ1 leu2Δ0 met15Δ0 ura3Δ0 spt21∆::KAN pESC-LEU2-pGAL-HHT2-HHF2* | This study |
| Sir2-FLAG | BY4741 | *MATa his3∆1 leu2∆0 met15∆0 ura3∆0 SIR2-3xFLAG:: KAN* | This study |
| Crm1-Myc | BY4741 | *MATa his3∆1 leu2∆0 met15∆0 ura3∆0 CRM1-13xMYC:: HIS3* | This study |
| H3/H4 KD Sir2-FLAG | BY4741 | *MATa his3∆1 leu2∆0 met15∆0 ura3∆0 hhf2-hht2::MET15 hhf1-hht1::LEU2, pDM9-HHT1-HHF1-URA SIR2-3xFLAG:: KAN* | This study |
| H3/H4 KD Crm1-Myc | BY4741 | *MATa his3∆1 leu2∆0 met15∆0 ura3∆0 hhf2-hht2::MET15 hhf1-hht1::LEU2, pDM9-HHT1-HHF1-URA CRM1-13xMYC:: HIS3* | This study |
| Sir2Y163P-FLAG | BY4741 | *MATa his3∆1 leu2∆0 met15∆0 ura3∆0 SIR2Y163P-3xFLAG:: KAN* | This study |
| H3/H4 KD Sir2Y163P-FLAG | BY4741 | *MATa his3∆1 leu2∆0 met15∆0 ura3∆0 hhf2-hht2::MET15 hhf1-hht1::LEU2, pDM9-HHT1-HHF1-URA* Sir2Y163P*-3xFLAG:: KAN* | This study |
| Eno2-FLAG | BY4741 | *MATa his3∆1 leu2∆0 met15∆0 ura3∆0 ENO2-3xFLAG:: KAN* | This study |
| Pgi1-FLAG | BY4741 | *MATa his3∆1 leu2∆0 met15∆0 ura3∆0 PGI1-3xFLAG:: KAN* | This study |
| WT GFP-ATG8 | BY4741 | *MATa his3∆1 leu2∆0 met15∆0 ura3∆0 GFP-ATG8::URA3* | This study |
| H3/H4 KD  GFP-ATG8 | BY4741 | *MATa his3∆1 leu2∆0 met15∆0 ura3∆0 hhf2-hht2::MET15 hhf1-hht1::LEU2, pDM9-HHT1-HHF1-URA GFP-ATG8::URA3* | This study |
| *set1∆* GFP-ATG8 | BY4741 | *MATa his3∆1 leu2∆0 met15∆0 ura3∆0 set1∆::KAN GFP-ATG8::URA3* | This study |
| *spp1∆* GFP-ATG8 | BY4741 | *MATa his3∆1 leu2∆0 met15∆0 ura3∆0 spp1∆::KAN GFP-ATG8::URA3* | This study |
| *spt21∆* GFP-ATG8 | BY4741 | *MATa his3∆1 leu2∆0 met15∆0 ura3∆0 spt21∆::KAN GFP-ATG8::URA3* | This study |
| *hir1∆* GFP-ATG8 | BY4741 | *MATa his3∆1 leu2∆0 met15∆0 ura3∆0 hir1∆::KAN GFP-ATG8::URA3* | This study |
| *hir2∆* GFP-ATG8 | BY4741 | *MATa his3∆1 leu2∆0 met15∆0 ura3∆0 hir2∆::KAN GFP-ATG8::URA3* | This study |
