# Supplementary material for: Regulation of telomere silencing by the core histones–autophagy–Sir2 axis
Source: Life Sci Alliance. 2022 Dec 30;6(3):e202201614. doi: 10.26508/lsa.202201614 (PMC9806677; doi:10.26508/lsa.202201614)
Supplement: Supplementary file 10 [file LSA-2022-01614_TableS2.docx]

**Supplemental Table 2 List of oligonucleotides used in this study**

| **Gene name** | **Sequence** |
| --- | --- |
| **ChIP-qPCR** | |
| *TEL VIR 1kb* | GTTATGTTAGAGATAACTGTGAG |
|  | GCTTGTTAACTCTCCGACAG |
| *TEL VIR 2.5kb* | GCAATGAATCTTCGGTGCTTGG |
|  | CCATACCAATATCAACTTCACGG |
| *TEL VIR 5kb* | CCCCGCCTTTGAAGATTGTCCC |
|  | CGAGACCCACTTGTATTCTTAGTGC |
| *TEL VIR 7.5kb* | CCTCTATAGGACCTGTCTCATGG |
|  | GGAAGTCTACACTAATAGCTATG |
| *TEL VIR 30.5kb* | CAACTCACCCAATCCGTCTAG |
|  | TCCAACTCAGCAATAGCGTC |
| *TEL VIR 64.8kb* | AAGGGTTTTGGGTTTGTTCAC |
|  | TCCAATTGAGAGTCACGTTCC |
| *PHO11* | GACAAAATCGGAACTCAAACGG |
|  | TCTTTCACCGTGTCTACCAAC |
| *SOR2* | TCATGAATCAAGCGGACAGG |
|  | GTTCAATAGCAACACGGTCAC |
| *YCR102C* | GTTGCCTACAAATCACCCAATG |
|  | AGGTTCAAGCCCAAGTTATAGG |
| *THI12* | ACCAATCCTTCCGATGTCAC |
|  | AAAGAGGCAACAGAGGTCAC |
| *YDL241W* | CATGGCTTCACAAATGCGAG |
|  | TTCTGTACCTTTCCCTTGCAG |
| *SOR1* | GTAGTTCTAGAGAAAGTCGGCG |
|  | GGCGCCTTCAATATGTACTTACC |
| *SEO1* | GGTTTAGAGGGATGGAGATGG |
|  | TCCTCGCCAACCTAATTTCATC |
| *PYK1* | CCCAATCCCACCAAACCAC |
|  | TTCTACCAGCGGAGATGACCTT |
| *KRE1* | CTGTGTCCTCGTCTGTATCTTC |
|  | GAGGTTCTGTGAAGTCTGTGG |
| **qRT-PCR** |  |
| *ACTIN* | CTGTCGAGAGATTTCTCTTTTACC |
|  | GCCCCTATTTATTCCAATAATATCG |
| *GAPDH* | CGGTAGATACGCTGGTGAAGTTTC |
|  | TGGAAGATGGAGCAGTGATAACAAC |
| *HHT2* | GCCCCAAGAAAACAATTAGCC |
|  | CTCTCAAGGCAACAGTACCTG |
| *HHF2* | TGGTGTCAAGCGTATTTCTGG |
|  | ACAGTCTTTCTCTTGGCGTG |
| *HTA2* | AGCTGGTTTAACATTCCCAGTT |
|  | GCAGTTAGATAGACTGGAGCAC |
| *HTB2* | GATTGATCTTACCTGGTGAATTGGCTAAA |
|  | GGCTTGAGTAGAGGAGGAGTAT |
| *HHT1* | CTGCCATTCACGCCAAGC |
|  | ATGATCTTTCACCTCTTAATCTTCTAGCC |
| *HHF1* | AAGAGATAACATCCAAGGTATTACTAAGCC |
|  | CAAACCAGAAATACGCTTGACAC |
| *HTA1* | GGTTCTGGTGCTCCAGTCTAC |
|  | TCTTCTTGTTATCCCTAGCAGCAT |
| *HTB1* | AGAGAAGCAAGGCTAGAAAGGA |
|  | GGAAATACCAGTGTCAGGGTG |
| *PHO11* | GACAAAATCGGAACTCAAACGG |
|  | TCTTTCACCGTGTCTACCAAC |
| *SOR2* | TCATGAATCAAGCGGACAGG |
|  | GTTCAATAGCAACACGGTCAC |
| *YCR102C* | GTTGCCTACAAATCACCCAATG |
|  | AGGTTCAAGCCCAAGTTATAGG |
| *THI12* | ACCAATCCTTCCGATGTCAC |
|  | AAAGAGGCAACAGAGGTCAC |
| *YDL241W* | CATGGCTTCACAAATGCGAG |
|  | TTCTGTACCTTTCCCTTGCAG |
| *SOR1* | GTAGTTCTAGAGAAAGTCGGCG |
|  | GGCGCCTTCAATATGTACTTACC |
| *SEO1* | GGTTTAGAGGGATGGAGATGG |
|  | TCCTCGCCAACCTAATTTCATC |
| *COS8* | CCGTTCTACCTCAAGATGTTTTCCG |
|  | CCAGGAACAGGACAAGAAGTGAAAC |
| *SIR2* | CCTCGAACTTCCACTATGCC |
|  | TGAGAACGCCATATGAGTTAAGG |
| *PYK1* | CCCAATCCCACCAAACCAC |
|  | TTCTACCAGCGGAGATGACCTT |
| *KRE1* | CTGTGTCCTCGTCTGTATCTTC |
|  | GAGGTTCTGTGAAGTCTGTGG |
| *PMA1* | AAGATCACCCAATCCCAGAAG |
|  | ACCCAAGATTTCCCAGTGAC |
| *PAB1* | AAGGGTTTTGGGTTTGTTCAC |
|  | TCCAATTGAGAGTCACGTTCC |
