# Supplementary material for: Regulation of telomere silencing by the core histones–autophagy–Sir2 axis
Source: Life Sci Alliance. 2022 Dec 30;6(3):e202201614. doi: 10.26508/lsa.202201614 (PMC9806677; doi:10.26508/lsa.202201614)
Supplement: Supplementary file 11 [file LSA-2022-01614_TableS3.docx]

**Supplemental Table 3 List of antibodies used in this study**

| **Antibody** | **Source** | **Identifier** |
| --- | --- | --- |
| Anti-GAPDH | Proteintech | 10494-1-AP |
| Anti-Actin | Proteintech | 20536-1-AP |
| Anti-Pgk1 | Molecular Probe | A-6457 |
| Anti-Tubulin | Abcam | ab6160 |
| Anti-FLAG | Abclonal | AE024 |
| Anti-Histone H3 | CST | 9715S |
| Anti-Histone H4 | Abclonal | A21676 |
| Anti-Histone H2A | Abclonal | E23363 |
| Anti-Histone H2B | CST | 12364S |
| Anti-H2A.Z | Abcam | Ab4626 |
| Anti-H3K4me3 | Abclonal | A2357 |
| Anti-Sir2 | Abclonal | E16590 |
| Anti-Sir3 | Santa Cruz Biotecnology | sc-28552 |
| Anti-GFP | Proteintech | 66002-1 |
| Anti-Myc | Proteintech | 60003-2 |
