# Supplementary material for: Regulation of telomere silencing by the core histones–autophagy–Sir2 axis
Source: Life Sci Alliance. 2022 Dec 30;6(3):e202201614. doi: 10.26508/lsa.202201614 (PMC9806677; doi:10.26508/lsa.202201614)
Supplement: Supplementary file 12 [file LSA-2022-01614_TableS4.docx]

**Supplemental Table 4 List of genes near telomeres in the indicated mutants**

|  |  | ***spt21****∆* **up-regulated** |  | ***spt21****∆* **down-regulated** |  |
| --- | --- | --- | --- | --- | --- |
| **Total genes** | **Distance from telomeres (kb)** | **Number of genes** | **% of genes** | **Number of genes** | **% of genes** |
| 180 | 10 | 42 | 23.33333333 | 3 | 1.666666667 |
| 150 | 20 | 34 | 22.66666667 | 1 | 0.666666667 |
| 156 | 30 | 22 | 14.1025641 | 1 | 0.641025641 |
| 146 | 40 | 14 | 9.589041096 | 0 | 0 |
| 196 | 50 | 21 | 10.71428571 | 0 | 0 |
| 178 | 60 | 14 | 7.865168539 | 0 | 0 |
|  | Whole-genome | 591 | 8.293572832 | 12 | 0.168397418 |

|  |  | **7.5hr up-regulated** |  | **7.5hr down-regulated** |  |
| --- | --- | --- | --- | --- | --- |
| **Total genes** | **Distance from telomeres (kb)** | **Number of genes** | **% of genes** | **Number of genes** | **% of genes** |
| 180 | 10 | 95 | 52.77777778 | 4 | 2.222222222 |
| 150 | 20 | 70 | 46.66666667 | 6 | 4 |
| 156 | 30 | 35 | 22.43589744 | 4 | 2.564102564 |
| 146 | 40 | 32 | 21.91780822 | 3 | 2.054794521 |
| 196 | 50 | 40 | 20.40816327 | 2 | 1.020408163 |
| 178 | 60 | 30 | 16.85393258 | 1 | 0.561797753 |
|  | Whole-genome | 851 | 11.94218355 | 89 | 1.248947516 |

|  |  | **24hr up-regulated** |  | **24hr down-regulated** |  |
| --- | --- | --- | --- | --- | --- |
| **Total genes** | **Distance from telomeres (kb)** | **Number of genes** | **% of genes** | **Number of genes** | **% of genes** |
|  |  |  |  |  |  |
| 180 | 10 | 105 | 58.33333333 | 5 | 2.777777778 |
| 150 | 20 | 100 | 66.66666667 | 0 | 0 |
| 156 | 30 | 82 | 52.56410256 | 1 | 0.641025641 |
| 146 | 40 | 76 | 52.05479452 | 0 | 0 |
| 196 | 50 | 67 | 34.18367347 | 3 | 1.530612245 |
| 178 | 60 | 57 | 32.02247191 | 2 | 1.123595506 |

|  |  | **48hr up-regulated** |  | **48hr down-regulated** |  |
| --- | --- | --- | --- | --- | --- |
| **Total genes** | **Distance from telomeres (kb)** | **Number of genes** | **% of genes** | **Number of genes** | **% of genes** |
| 180 | 10 | 151 | 83.88888889 | 2 | 1.111111111 |
| 150 | 20 | 117 | 78 | 0 | 0 |
| 156 | 30 | 104 | 66.66666667 | 4 | 2.564102564 |
| 146 | 40 | 100 | 68.49315068 | 1 | 0.684931507 |
| 196 | 50 | 91 | 46.42857143 | 8 | 4.081632653 |
| 178 | 60 | 86 | 48.31460674 | 8 | 4.494382022 |
|  | Whole-genome | 3463 | 48.59668818 | 285 | 3.999438675 |

|  |  | **H3R2A up-regulated** |  | **H3R2A down-regulated** |  |
| --- | --- | --- | --- | --- | --- |
| **Total genes** | **Distance from telomeres (kb)** | **Number of genes** | **% of genes** | **Number of genes** | **% of genes** |
| 180 | 10 | 6 | 3.333333333 | 10 | 5.555555556 |
| 150 | 20 | 9 | 6 | 9 | 6 |
| 156 | 30 | 18 | 11.53846154 | 13 | 8.333333333 |
| 146 | 40 | 25 | 17.12328767 | 11 | 7.534246575 |
| 196 | 50 | 21 | 10.71428571 | 11 | 5.612244898 |
| 178 | 60 | 23 | 12.92134831 | 8 | 4.494382022 |
|  | Whole-genome | 641 | 8.99522874 | 422 | 5.921975863 |

|  |  | **H3R17A up-regulated** |  | **H3R17A down-regulated** |  |
| --- | --- | --- | --- | --- | --- |
| **Total genes** | **Distance from telomeres (kb)** | **Number of genes** | **% of genes** | **Number of genes** | **% of genes** |
| 180 | 10 | 5 | 2.777777778 | 6 | 3.333333333 |
| 150 | 20 | 7 | 4.666666667 | 4 | 2.666666667 |
| 156 | 30 | 8 | 5.128205128 | 0 | 0 |
| 146 | 40 | 3 | 2.054794521 | 4 | 2.739726027 |
| 196 | 50 | 2 | 1.020408163 | 0 | 0 |
| 178 | 60 | 1 | 0.561797753 | 1 | 0.561797753 |
|  | Whole-genome | 89 | 1.248947516 | 82 | 1.150715689 |

|  |  | **H3R40A up-regulated** |  | **H3R40A down-regulated** |  |
| --- | --- | --- | --- | --- | --- |
| **Total genes** | **Distance from telomeres (kb)** | **Number of genes** | **% of genes** | **Number of genes** | **% of genes** |
| 180 | 10 | 5 | 2.777777778 | 6 | 3.333333333 |
| 150 | 20 | 3 | 2 | 3 | 2 |
| 156 | 30 | 3 | 1.923076923 | 2 | 1.282051282 |
| 146 | 40 | 4 | 2.739726027 | 0 | 0 |
| 196 | 50 | 3 | 1.530612245 | 0 | 0 |
| 178 | 60 | 2 | 1.123595506 | 1 | 0.561797753 |
|  | Whole-genome | 98 | 1.37524558 | 32 | 0.449059781 |

|  |  | **H3R49A up-regulated** |  | **H3R49A down-regulated** |  |
| --- | --- | --- | --- | --- | --- |
| **Total genes** | **Distance from telomeres (kb)** | **Number of genes** | **% of genes** | **Number of genes** | **% of genes** |
| 180 | 10 | 21 | 11.66666667 | 5 | 2.777777778 |
| 150 | 20 | 18 | 12 | 8 | 5.333333333 |
| 156 | 30 | 16 | 10.25641026 | 12 | 7.692307692 |
| 146 | 40 | 15 | 10.2739726 | 19 | 13.01369863 |
| 196 | 50 | 16 | 8.163265306 | 11 | 5.612244898 |
| 178 | 60 | 22 | 12.35955056 | 13 | 7.303370787 |
|  | Whole-genome | 666 | 9.346056694 | 649 | 9.107493685 |

|  |  | **H3R72A up-regulated** |  | **H3R72A down-regulated** |  |
| --- | --- | --- | --- | --- | --- |
| **Total genes** | **Distance from telomeres (kb)** | **Number of genes** | **% of genes** | **Number of genes** | **% of genes** |
| 180 | 10 | 14 | 7.777777778 | 2 | 1.111111111 |
| 150 | 20 | 3 | 2 | 4 | 2.666666667 |
| 156 | 30 | 5 | 3.205128205 | 3 | 1.923076923 |
| 146 | 40 | 9 | 6.164383562 | 2 | 1.369863014 |
| 196 | 50 | 1 | 0.510204082 | 1 | 0.510204082 |
| 178 | 60 | 3 | 1.685393258 | 1 | 0.561797753 |
|  | Whole-genome | 116 | 1.627841706 | 126 | 1.768172888 |

|  |  | **H4K44A up-regulated** |  | **H4K44A down-regulated** |  |
| --- | --- | --- | --- | --- | --- |
| **Total genes** | **Distance from telomeres (kb)** | **Number of genes** | **% of genes** | **Number of genes** | **% of genes** |
| 180 | 10 | 24 | 13.33333333 | 6 | 3.333333333 |
| 150 | 20 | 23 | 15.33333333 | 10 | 6.666666667 |
| 156 | 30 | 18 | 11.53846154 | 14 | 8.974358974 |
| 146 | 40 | 25 | 17.12328767 | 16 | 10.95890411 |
| 196 | 50 | 18 | 9.183673469 | 19 | 9.693877551 |
| 178 | 60 | 19 | 10.6741573 | 16 | 8.988764045 |
|  | Whole-genome | 785 | 11.01599775 | 654 | 9.177659276 |

|  |  | **H4R55A up-regulated** |  | **H4R55A down-regulated** |  |
| --- | --- | --- | --- | --- | --- |
| **Total genes** | **Distance from telomeres (kb)** | **Number of genes** | **% of genes** | **Number of genes** | **% of genes** |
| 180 | 10 | 20 | 11.11111111 | 11 | 6.111111111 |
| 150 | 20 | 26 | 17.33333333 | 10 | 6.666666667 |
| 156 | 30 | 20 | 12.82051282 | 21 | 13.46153846 |
| 146 | 40 | 18 | 12.32876712 | 14 | 9.589041096 |
| 196 | 50 | 20 | 10.20408163 | 12 | 6.12244898 |
| 178 | 60 | 19 | 10.6741573 | 12 | 6.741573034 |
|  | Whole-genome | 807 | 11.32472635 | 446 | 6.258770699 |

|  |  | **H3D77A up-regulated** |  | **H3D77A down-regulated** |  |
| --- | --- | --- | --- | --- | --- |
| **Total genes** | **Distance from telomeres (kb)** | **Number of genes** | **% of genes** | **Number of genes** | **% of genes** |
| 180 | 10 | 3 | 1.666666667 | 23 | 12.77777778 |
| 150 | 20 | 0 | 0 | 9 | 6 |
| 156 | 30 | 2 | 1.282051282 | 0 | 0 |
| 146 | 40 | 3 | 2.054794521 | 2 | 1.369863014 |
| 196 | 50 | 1 | 0.510204082 | 2 | 1.020408163 |
| 178 | 60 | 1 | 0.561797753 | 0 | 0 |
|  | Whole-genome | 59 | 0.827953971 | 78 | 1.094583216 |

|  |  | ***set1****∆*  **up-regulated** |  | ***set1****∆* **down-regulated** |  |
| --- | --- | --- | --- | --- | --- |
| **Total genes** | **Distance from telomeres (kb)** | **Number of genes** | **% of genes** | **Number of genes** | **% of genes** |
| 180 | 10 | 6 | 3.333333333 | 5 | 2.777777778 |
| 150 | 20 | 10 | 6.666666667 | 6 | 4 |
| 156 | 30 | 15 | 9.615384615 | 4 | 2.564102564 |
| 146 | 40 | 14 | 9.589041096 | 5 | 3.424657534 |
| 196 | 50 | 12 | 6.12244898 | 3 | 1.530612245 |
| 178 | 60 | 12 | 6.741573034 | 5 | 2.808988764 |
|  | Whole-genome | 325 | 4.560763402 | 115 | 1.613808588 |
